# Supplementary material for: Quantum-walk search in motion
Source: Sci Rep. 2024 Feb 2;14:2815. doi: 10.1038/s41598-024-51709-0 (PMC10837460; doi:10.1038/s41598-024-51709-0)
Supplement: Supplementary file 1 — Supplementary Information 1. [file 41598_2024_51709_MOESM1_ESM.pdf]

Supplementary Material

Flip-Flop Shift Operator The flip-flop shift operator for open boundary condition is given by sum of interior term

$$\begin{aligned}
S_{\text{int}} = |\downarrow\rangle\langle\uparrow| \otimes \sum_{x,y<\sqrt{N}-1} |x,y+1\rangle\langle x,y| + |\uparrow\rangle\langle\downarrow| \otimes \sum_{x,y>0} |x,y-1\rangle\langle x,y| \\
+ |\leftarrow\rangle\langle\rightarrow| \otimes \sum_{x<\sqrt{N}-1,y} |x+1,y\rangle\langle x,y| + |\rightarrow\rangle\langle\leftarrow| \otimes \sum_{x>0,y} |x-1,y\rangle\langle x,y| \\
= |\downarrow\rangle\langle\uparrow| \otimes I \otimes \sum_{y<\sqrt{N}-1} |y+1\rangle\langle y| + |\uparrow\rangle\langle\downarrow| \otimes I \otimes \sum_{y>0} |y-1\rangle\langle y| \\
+ |\leftarrow\rangle\langle\rightarrow| \otimes \sum_{x<\sqrt{N}-1} \otimes I|x+1\rangle\langle x| \otimes I + |\rightarrow\rangle\langle\leftarrow| \otimes \sum_{x>0} |x-1\rangle\langle x| \otimes I
\end{aligned} \tag{1}$$

and boundary (exterior) term

$$\begin{aligned}
S_{\text{ext}} = |\uparrow\rangle\langle\uparrow| \otimes \sum_{x,y=\sqrt{N}-1} |x,y\rangle\langle x,y| + |\downarrow\rangle\langle\downarrow| \otimes \sum_{x,y=0} |x,y\rangle\langle x,y| \\
+ |\rightarrow\rangle\langle\rightarrow| \otimes \sum_{x=\sqrt{N}-1,y} |x,y\rangle\langle x,y| + |\leftarrow\rangle\langle\leftarrow| \otimes \sum_{x=0,y} |x,y\rangle\langle x,y| \\
= |\uparrow\rangle\langle\uparrow| \otimes I \otimes |\sqrt{N}-1\rangle\langle\sqrt{N}-1| + |\downarrow\rangle\langle\downarrow| \otimes I \otimes |0\rangle\langle 0| \\
+ |\rightarrow\rangle\langle\rightarrow| \otimes |\sqrt{N}-1\rangle\langle\sqrt{N}-1| \otimes I + |\leftarrow\rangle\langle\leftarrow| \otimes |0\rangle\langle 0| \otimes I
\end{aligned} \tag{2}$$

as $S_{\text{o}} = S_{\text{int}} + S_{\text{ext}}$. The form of S_{int} and S_{ext} is chosen so that the shift operator S_{o} is unitary i.e. $S_{\text{o}}(S_{\text{o}})^\dagger = (S_{\text{o}})^\dagger S_{\text{o}} = I$. To verify this, consider

$$\begin{aligned}
S_{\text{o}}(S_{\text{o}})^\dagger &= (S_{\text{int}} + S_{\text{ext}})(S_{\text{int}}^\dagger + S_{\text{ext}}^\dagger) \\
&= S_{\text{int}}S_{\text{int}}^\dagger + S_{\text{int}}S_{\text{ext}}^\dagger + S_{\text{ext}}S_{\text{int}}^\dagger + S_{\text{ext}}S_{\text{ext}}^\dagger \\
&= S_{\text{int}}S_{\text{int}}^\dagger + S_{\text{ext}}S_{\text{ext}}^\dagger
\end{aligned}$$

where we used $S_{\text{int}}S_{\text{ext}}^\dagger = 0 = S_{\text{ext}}S_{\text{int}}^\dagger$, which follows from the orthonormality of the position basis. The two terms can easily be found using the explicit form of S_{int} and S_{ext}.

$$\begin{aligned}
S_{\text{int}}S_{\text{int}}^\dagger = |\downarrow\rangle\langle\downarrow| \otimes I \otimes \sum_{y=1}^{\sqrt{N}-1} |y\rangle\langle y| + |\uparrow\rangle\langle\uparrow| \otimes I \otimes \sum_{y=0}^{\sqrt{N}-2} |y\rangle\langle y| \\
+ |\leftarrow\rangle\langle\leftarrow| \otimes \sum_{x=1}^{\sqrt{N}-1} |x\rangle\langle x| \otimes I + |\rightarrow\rangle\langle\rightarrow| \otimes \sum_{x=0}^{\sqrt{N}-2} |x\rangle\langle x| \otimes I \\
S_{\text{ext}}S_{\text{ext}}^\dagger = |\uparrow\rangle\langle\uparrow| \otimes I \otimes |\sqrt{N}-1\rangle\langle\sqrt{N}-1| + |\downarrow\rangle\langle\downarrow| \otimes I \otimes |0\rangle\langle 0| \\
+ |\rightarrow\rangle\langle\rightarrow| \otimes |\sqrt{N}-1\rangle\langle\sqrt{N}-1| \otimes I + |\leftarrow\rangle\langle\leftarrow| \otimes |0\rangle\langle 0| \otimes I
\end{aligned}$$

Adding the two terms together, we get $S_{\text{int}}S_{\text{int}}^\dagger + S_{\text{ext}}S_{\text{ext}}^\dagger = S_{\text{o}}(S_{\text{o}})^\dagger = I$. As required. Note that this unitarity breaks if we consider the exterior term to be

$$S_{\text{ext}} = |\downarrow\rangle\langle\uparrow| \otimes \sum_{x,y=\sqrt{N}-1} |x,y\rangle\langle x,y| + |\uparrow\rangle\langle\downarrow| \otimes \sum_{x,y=0} |x,y\rangle\langle x,y| + |\leftarrow\rangle\langle\rightarrow| \otimes \sum_{x=\sqrt{N}-1,y} |x,y\rangle\langle x,y| + |\rightarrow\rangle\langle\leftarrow| \otimes \sum_{x=0,y} |x,y\rangle\langle x,y|\,,$$

which gives

$$S_{\text{ext}}S_{\text{ext}}^{\dagger} = |\downarrow\rangle\langle\downarrow| \otimes I \otimes |\sqrt{N}-1\rangle\langle\sqrt{N}-1| + |\uparrow\rangle\langle\uparrow| \otimes I \otimes |0\rangle\langle 0| + |\leftarrow\rangle\langle\leftarrow| \otimes |\sqrt{N}-1\rangle\langle\sqrt{N}-1| \otimes I + |\rightarrow\rangle\langle\rightarrow| \otimes |0\rangle\langle 0| \otimes I\,.$$

It is easier to note that $S_{\text{int}}S_{\text{int}}^{\dagger} + S_{\text{ext}}S_{\text{ext}}^{\dagger} = S_{\text{o}}(S_{\text{o}})^{\dagger} \neq I$.

Quantum-walk search algorithm Here, we will give numerical results for the conventional QWSA in two-dimentional lattice with open and periodic boundary conditions. The marked points as chosen same as in the main text i.e. $\{(6,8),(8,9),(12,5),(15,5)\}$.

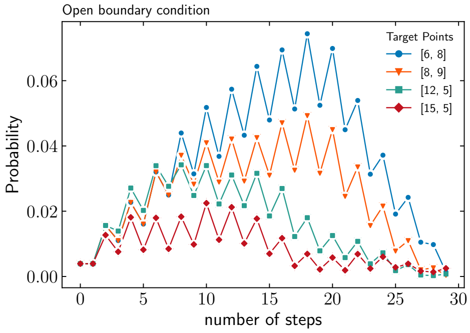

(a) $2d$ open grid

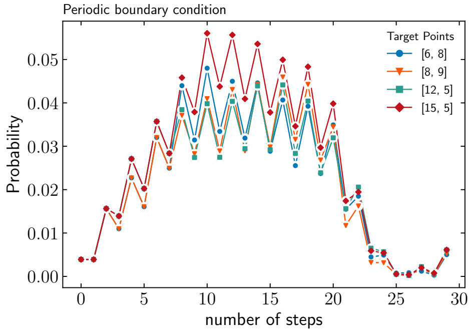

(b) Torus

Supplementary Figure S1: Amplification of marked nodes with steps for conventional QWSA in the case of **(a)** open grid and **(b)** torus.

In Figure 1, we show the probability of finding a labeled marked node as a function of the number of steps taken by the algorithm.
