# Supplementary figures and images for: Quantum-walk search in motion
Source: Sci Rep. 2024 Feb 2;14:2815. doi: 10.1038/s41598-024-51709-0 (PMC10837460; doi:10.1038/s41598-024-51709-0)

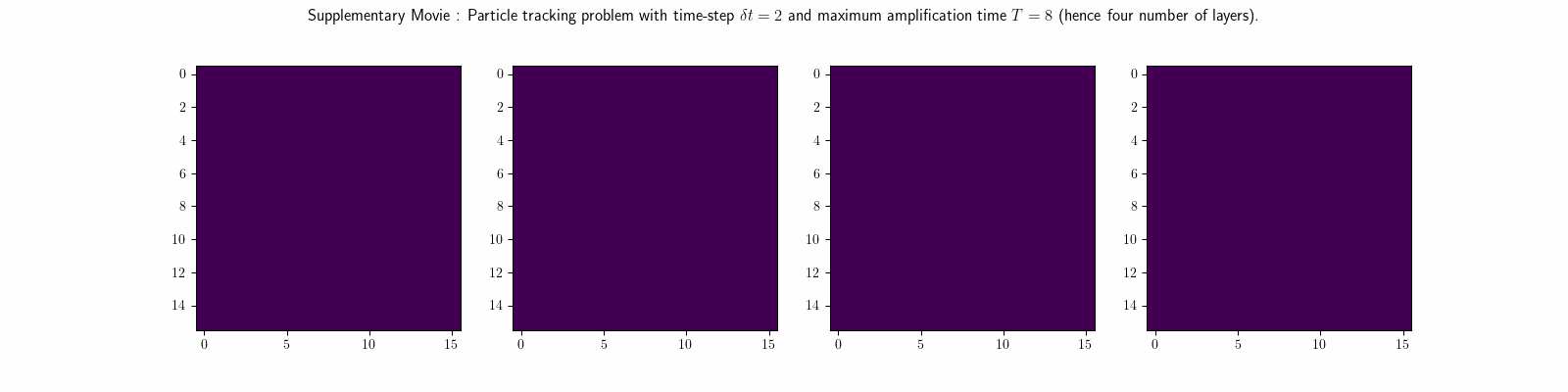

Supplement: Supplementary file 2 — Supplementary Information 2. [file 41598_2024_51709_MOESM2_ESM.gif]
